# Effect of [10]-Gingerol on [Ca^2+^]_i_ and Cell Death in Human Colorectal Cancer Cells

**DOI:** 10.3390/molecules14030959

**Published:** 2009-03-02

**Authors:** Chung-Yi Chen, Yi-Wen Li, Soong-Yu Kuo

**Affiliations:** Department of Medical Technology, School of Medicine and Health Sciences, Fooyin University, Kaohsiung County 83101 Taiwan; E-mails: xx377@mail.fy.edu.tw (C-Y.C.), bestgat@yahoo.com.tw ( Y-W.L.)

**Keywords:** Ca^2+^, [10]-Gingerol, L-type Ca^2+^ channel blockers, SW480 cells, Thapsigargin

## Abstract

The effect of [10]-gingerol on cytosol free Ca^2+^ concentration ([Ca^2+^]_i_) and viability is large unknown. This study examines the early signaling effects of [10]-gingerol on human colorectal cancer cells. It was found that this compound caused a slow and sustained rise of [Ca^2+^]_i_ in a concentration-dependent manner. [10]-Gingerol also induced a [Ca^2+^]_i_ rise when extracellular Ca^2+^ was removed, but the magnitude was reduced by 38%. In a Ca^2+^-free medium, the [10]-gingerol-induced [Ca^2+^]_i_ rise was partially abolished by depleting stored Ca^2+^ with thapsigargin (an endoplasmic reticulum Ca^2+^ pump inhibitor). The elevation of [10]-gingerol-caused [Ca^2+^]_i_ in a Ca^2+^-containing medium was not affected by modulation of protein kinase C activity. The [10]-gingerol-induced Ca^2+^ influx was insensitive to L-type Ca^2+^ channel blockers. At concentrations of 10-100 μM, [10]-gingerol killed cells in a concentration-dependent manner. These findings suggest that [10]-gingerol induces [Ca^2+^]_i_ rise by causing Ca^2+^ release from the endoplasmic reticulum and Ca^2+^ influx from non-L-type Ca^2+^ channels in SW480 cancer cells.

## Introduction

Ginger (*Zingiber officinale* L. Zingiberaceae) is a common condiment for various foods and beverages. Ginger also has a long history of use in traditional medicine. The underground stems or rhizomes of this plant have been used as a medicine in East Asian, Indian, and Arabic herbal traditions since ancient times [1]. In mainland China, the rhizomes of ginger have been used in oriental medicine for the treatment of the common cold, disorders of the gastrointestinal tract, neuralgia, rheumatism, colic, and motion discomfort [2,3]. The non-volatile pungent ingredients from ginger include gingerol, shogaol and zingerone. Recently, several population-based studies have shown that persons in Southeast Asian countries have a much lower risk of colon, gastrointestinal, prostate, breast, and other cancers than those in European and American countries [4]. It is believed that constituents of their diet may play important roles in cancer prevention. Indeed, some phenolic substances present in fruit and vegetables, and in medicinal plants, have potential cancer chemopreventive activities, as supported by both *in vitro* and *in vivo* in experiments [2,5,6,7]. These agents are known to have the ability to suppress the transformative, hyperproliferative, and inflammatory processes of carcinogenesis. 

**Figure 1:**
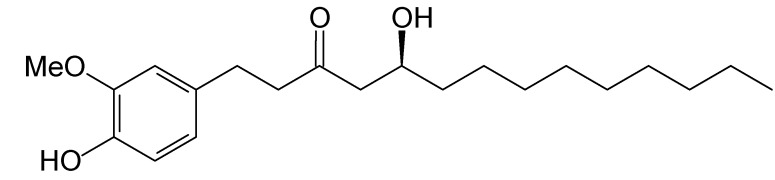
Structure of [10]-gingerol.

The phenolic compounds derived from ginger possess many interesting pharmacological and physiological activities. For example, [6]-gingerol [1-(4′-hydroxy-3′-methoxyphenyl)-5-hydroxy-3- decanone], the major pungent principle of ginger, has potential anti-inflammatory, antioxidant, anticarcinogenic, and antimutagenic activities [8,9,10]. Evidence indicates that [6]-gingerol exerts an inhibitory effect on DNA synthesis, also causes apoptosis in human promyelocytic leukemia (HL-60) cells [11]. *In vitro*, [6]-gingerol inhibited both the VEGF- and bFGF-induced proliferation of human endothelial cells and caused cell-cycle arrest in the G_1_ phase [12]. This compound also induced [Ca^2+^]_i_ elevation and was cytotoxic to canine renal cells [13]. In the case of [10]-gingerol (Figure 1), its effect on human promyelocytic leukemia (HL-60) cells is better than [6]-gingerol’s [11] and the activity of sarcoplasmic reticulum of Ca^2+^-ATPase could be stimulated by [10]-gingerol [14]. However, the detailed mechanism of [10]-gingerol’s anticarcinogenic effects is still unclear. 

The effect of [10]-gingerol on Ca^2+^ signaling and cytotoxicity in human colon cancer SW480 cells has not been explored. Colorectal cancer is the third most frequent and second most lethal in the United States [15]. Therefore, there is a need to search more effective chemotherapeutic agents that can be used to remedy the patients who have failed to respond under traditional chemotherapy. This study was performed to elucidate whether [10]-gingerol affects human colorectal tumorigenesis. Using fura-2 as a fluorescent Ca^2+^ indicator, we report for the first time that [10]-gingerol induced a significant and prolonged [Ca^2+^]_i_ increase and cytotoxicity in human colorectal cancer cells. The concentration-response relationship, the Ca^2+^ sources of the Ca^2+^ signal, and the role of protein kinase C in the signal have been investigated. 

## Results and Discussion

### Effect of [10]-gingerol on [Ca^2+^]_i_

**Figure 2:**
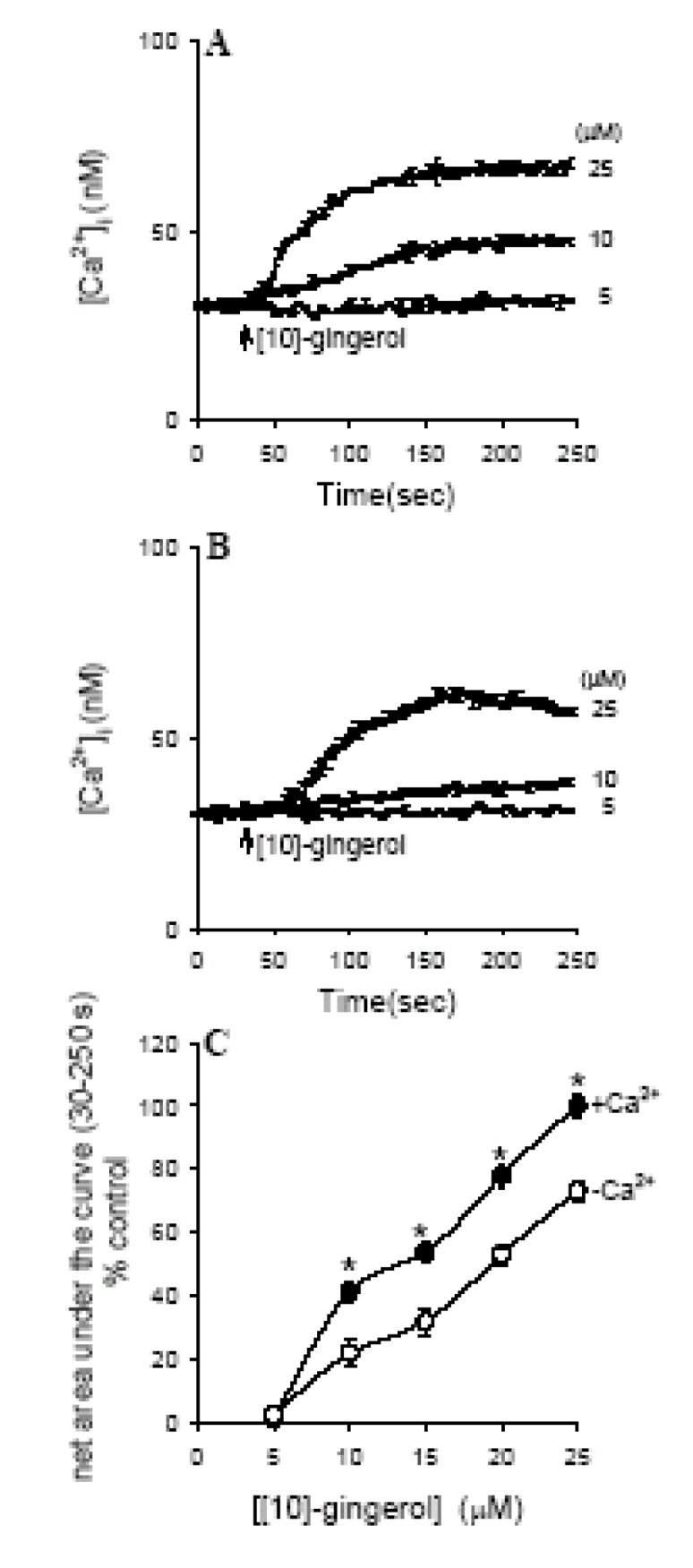
Effects of [10]-gingerol on [Ca^2+^]_i_ in SW480 cells. (A) Concentration-dependent effects of [10]-gingerol, with the concentration of the reagent indicated. Experiments were performed in Ca^2+^-containing medium. [10]-Gingerol was added at 30 sec and was present throughout the measurements for 250 sec. (B) Effect of extracellular Ca^2+^ removal on [10]-gingerol-induced [Ca^2+^]_i_ elevation. The concentration of [10]-Gingerol is indicated. (C) Concentration-response plots of [10]-gingerol-induced [Ca^2+^]_i _rises in Ca^2+^-containing medium (filled circles) and Ca^2+^-free medium (open circles). The data are presented as the percentage of control, which is the net [Ca^2+^]_i_ rise induced by 25 μM [10]-gingerol in Ca^2+^-containing medium. Data are mean SEM of five experiments (*p < 0.05 compared to open circles).

[10]-Gingerol at concentrations between 5-25 μM increased [Ca^2+^]_i _in a concentration- dependent manner in the presence of extracellular Ca^2+^. Figure 2A shows typical recordings of the [Ca ^2+^]_i _rise induced by 5-25 μM [10]-gingerol. 

At a concentration of 0.1 μM, [10]-gingerol had no effect on [Ca^2+^]_i_ (i.e., equivalent to baseline, 0 μM). The [Ca^2+^]_i_ rise induced by 5-25 μM [10]-gingerol comprised an immediate rise and a sustained phase in 250 sec. At a concentration of 25 μM, the [Ca^2+^]_i_ rise had a net value of 752nM at 250 sec. Figure 2C the [10]-gingerol-induced response.

### Effect of removing extracellular Ca^2+^ on [10]-gingerol-induced [Ca^2+^]_i_ signals

Experiments were performed to evaluate the relative contribution of extracellular Ca^2+^ entry and Ca^2+^ release from stores in the [10]-gingerol response. Figure 2B shows that removal of extracellular Ca^2+^ largely suppressed the [10]-gingerol-induced [Ca^2+^]_i_ elevation. The concentration-response relationship of [10]-gingerol-induced [Ca^2+^]_i _rise in the presence and absence of extracellular Ca^2+^ is shown in Figure 2C. Ca ^2+^ removal inhibited the [Ca^2+^]_i _rise caused by 25 μM [10]-gingerol by 38% as the maximum value (*n* = 5; *p* <0.05).

### Internal Ca^2+^ stores for [10]-gingerol-induced [Ca^2+^]_i_ Rises

**Figure 3:**
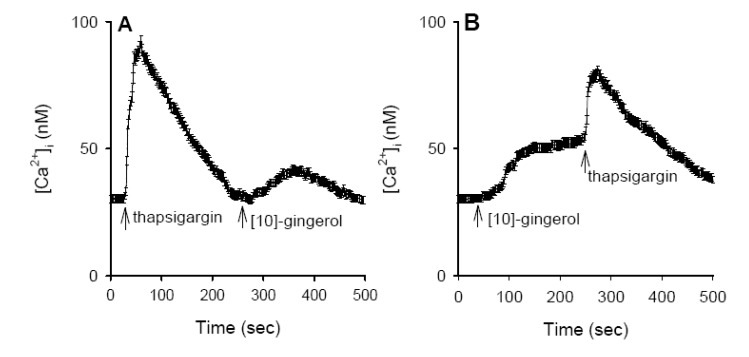
Intracellular sources of [10]-gingerol-induced [Ca^2+^]_i _elevation. Experiments were performed in Ca^2+^-free medium. Reagents were applied at the times indicated by arrows. (A) Thapsigargin (1 μM) and [10]-gingerol (25 μM) were added at 30 s and 250 s, respectively. (B) [10]-gingerol and thapsigargin were added at 30 s and 250 s, respectively. Data are means SEM of five experiments.

Previous reports have shown that the endoplasmic reticulum is a major Ca^2+^ store in the majority of cells [16]. Figure 3A shows that in Ca^2+^-free medium, application of 1 μM thapsigargin caused a [Ca^2+^]_i_ rise that comprised an initial increase and a gradual decay toward baseline. The net maximum [Ca^2+^]_i_ value was 96±4 nM (*n* = 5). After depleting the endoplasmic reticulum Ca^2+^ store with thapsigargin, addition of 25 μM [10]-gingerol did not induce a [Ca^2+^]_i_ rise, as shown in Figure 2B. Conversely, Figure 3B shows that after preincubation with [10]-gingerol (25 μM) for 250 sec, subsequent addition of 1 μM thapsigargin induced a [Ca^2+^]_i_ rise with a net value of 72±3 nM (*n* = 5), which was 25% (*p* < 0.05), smaller than the control thapsigargin response shown in Figure 3A.

### Lack of inhibitory effect of L-type Ca^2+^ entry blockers on Ca^2+^ release by [10]-gingerol

**Figure 4:**
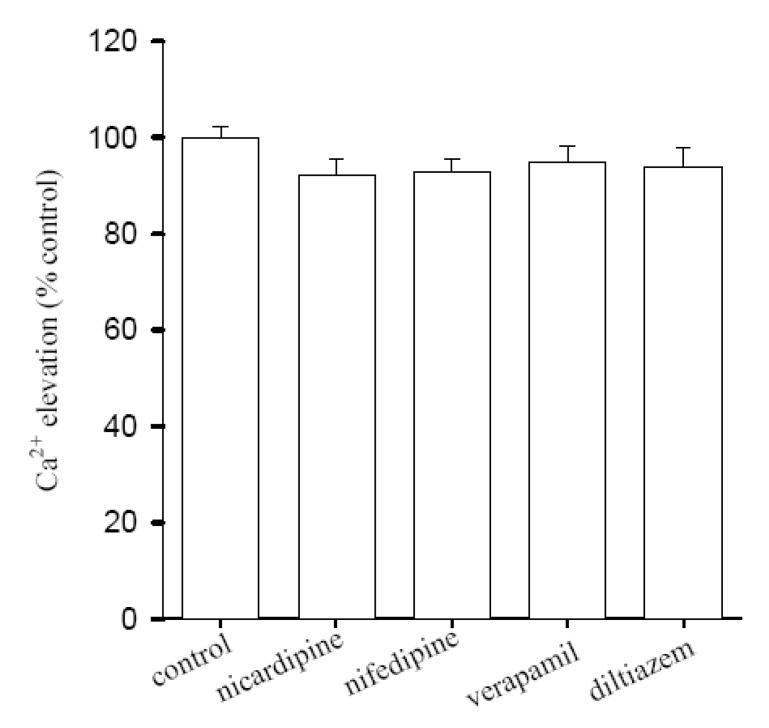
Effect of Ca^2+^ channel blockers on [10]-gingerol-induced [Ca^2+^]_i_ elevation. All experiments were performed in Ca^2+^-containing medium. The data are presented as the percentage of the control, which is the net area under the curve (30-250 sec) of the [Ca^2+^]_i_ rise induced by 25 μM [10]-gingerol. Data are means SEM of five experiments.

To explore the pathways underlying [10]-gingerol-induced Ca^2+^ entry, the effects of several L-type Ca^2+^ entry blockers on [10]-gingerol-induced [Ca^2+^]_i_ rise were evaluated. Figure 4 shows that in Ca^2+^-containing medium, pretreatment with 1 μM nicardipine did not inhibited 25 μM [10]-gingerol-induced [Ca^2+^]_i _elevation (*n* = 5; *p* < 0.05). Similarly, the Ca^2+^ influx was not affected by 1 μM diltiazem, nifedipine, or verapamil (*n* = 5).

### Regulation of [10]-gingerol-induced Ca^2+^ influx by Protein Kinase C

**Figure 5:**
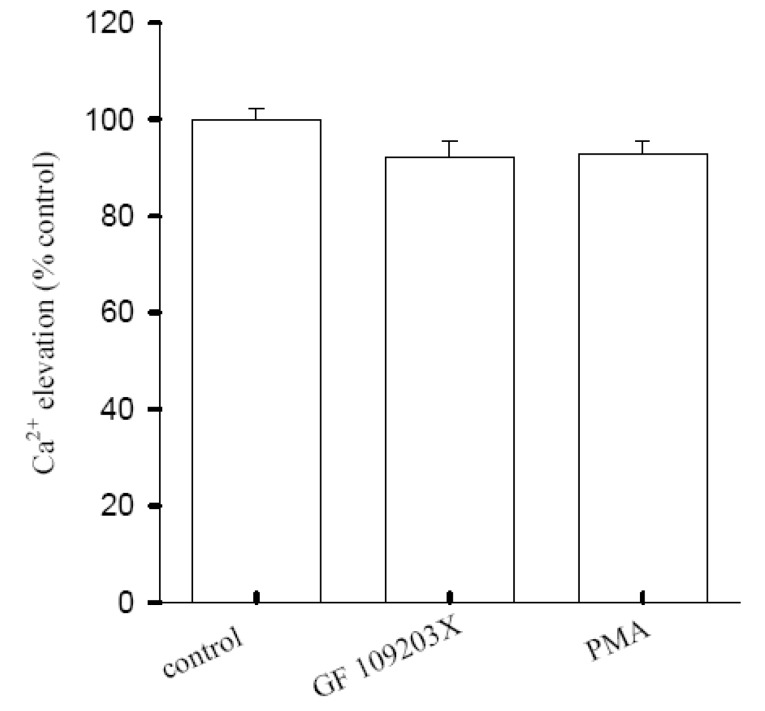
Effect of protein kinase C modulator on [10]-gingerol-induced [Ca^2+^]_i_ elevation. Experiments were performed in Ca^2+^-containing medium. PMA(10 nM) or GF 109203X (2 μM) was added 1 min prior to 25 μM 10-gingerol. Data are expressed as the percentage of control that is the net area under the curve of 25μM-[10]-gingerol-induced [Ca^2+^]i rise (30-250 sec interval), and are means SEM of five experiments.

A Ca^2+^ signal could be modulated by the activity of protein kinase C [17,18]. The role of protein kinase C in [10]-gingerol-induced [Ca^2+^]_i_ elevation was investigated. Figure 5 shows that the 25 μM [10]-gingerol-induced [Ca^2+^]_i _elevation was not altered by pretreatment with 10 nM phorbol myristate acetate (PMA, a protein kinase C activator) or 2 μM GF109203 X (a protein kinase C inhibitor) (*n* = 5).

### Effect of [10]-gingerol on Cell Viability

**Figure 6:**
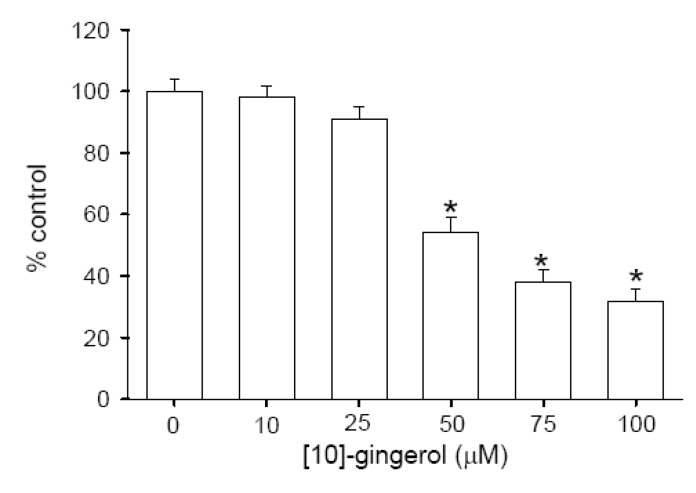
Cytotoxic effect of [10]-gingerol on human colorectal cancer SW480 cells. The cell viability was determined by the trypan blue exclusion (TBE) method. Data are expressed as the percentage of control ([10]-gingerol was absent).

Given the acute incubation with [10]-gingerol caused a substantial and lasting (Ca^2+^)_i_ response, and unregulated (Ca^2+^)_i_ rises can be linked to cytotoxicity [16,19], experiments were performed to examine the effect of 24 h incubation with [10]-gingerol on the viability of SW480 cells. Colon cancer cells were treated with 0-100 μM 24 h, and the trypan blue method was performed. In the exposure of 10-25 μM [10]-gingerol, cell viability was unchanged. However, at concentrations of 50-100 μM. [10]-gingerol decreased cell viability in a concentration-dependent manner (Figure 6; n=5).

This study is the first to investigate the effects of [10]-gingerol on [Ca^2+^]_i_ in colon cancer cells. The results obtained suggest that [10]-gingerol causes a significant concentration-dependent, sustained [Ca^2+^]_i_ rise in human colorectal SW480 cancer cells. In Ca^2+^-containing medium, the [Ca^2+^]_i_ rise induced by [10]-gingerol was sustained without a decay during the 5 min. of measurements. Sustained [Ca^2+^]_i_ elevations are thought to alter many cellular functions [20]. [10]-Gingerol may affect cell physiology significantly by changing Ca^2+ ^signaling and stimulating Ca^2+^-coupled bioactive molecules. The results show that the [Ca^2+^]_i_ rise was contributed to by both intracellular Ca^2+^ release and extracellular Ca^2+^ influx, because the signal was suppressed by removal of extracellular Ca^2+^.

Regarding the Ca^2+^ depositories of the [10]-gingerol response, the present data show that thapsigargin-sensitive endoplasmic reticulum store appears to play a crucial role because the [10]-gingerol-induced Ca^2+^ release was partly abolished by depletion of the endoplasmic reticulumic Ca^2+^ store with thapsigargin, and, conversely, pretreatment with [10]-gingerol also inhibited thapsigargin-induced Ca^2+^ release. The endoplasmic reticulum is one of the major Ca^2+^ stores where various proteins and lipids are synthesized and modified [21,22]. Perturbation of endoplasmic reticulum Ca^2+^ homeostasis, protein misfolding, or oxidative stress can lead to cell death [22,23]. Evidence reveals that reactive oxygen species (ROS) and the oxidation-reduction (redox) state play significant roles in many cytotoxic pathways caused by frequently used antitumor drugs or environmental toxicants [24]. A rise in [Ca^2+^]_i_ induced by oxidants may activate Ca^2+^-dependent enzymes such as proteases, nucleases, and phospholipases to facilitate mitochondrial oxidative stress leading to cytotoxicity [25,26]. Exactly how [10]-gingerol releases Ca^2+ ^stored in the endoplasmic reticulum is unclear, but the process seems to be independent on protein kiase C activity because suppression of this protein did not affect [10]-gingerol-induced Ca^2+^ release. Because [10]-gingerol and thapsigargin share the same Ca^2+^ stores, [10]-gingerol may very likely release Ca^2+^ in a manner similarly to thapsigargin by inhibiting endoplasmic reticulum Ca^2+^ pump. 

In Ca^2+^-free medium, the [10]-gingerol-induced [Ca2+]i elevation displayed a smaller [Ca2+]i increase throughout the measurement period of 250 sec. This suggests that Ca2+ influx contributed not only to the initial increase, but also to the prolonged phase of the [10]-gingerol-induced [Ca^2+^]_i_ signal in the Ca^2+^-containing medium. In non-excitable cells, a possible Ca^2+^ influx pathway is a store-operated Ca^2+^ entry, a process triggered by depletion of Ca^2+^ stores [27]. This possibility was not explored due to the lack of selective pharmacological inhibitors for this Ca^2+^ influx [28]. Thus, it remains possible that Ca^2+^ entry mechanisms other than depletion-activated channels may be important in Ca^2+^ influx in non-excitable cells. [10-]-gingerol is cytotoxic in several cell types including human A549, SK-OV-3, SK-MEL-2, and HCT15 tumor cells [34]. The present data indicate that [10]-gingerol at 50 μM or higher concentrations caused concentration-dependent cell death; whereas at lower concentrations it had no cytotoxic effects.

We are the first to demonstrate that in the presence of 10-100 μM [10]-gingerol, the viability of SW480 cells decreased in a concentration-dependent manner. Ca^2+^ overloading is known to initiate processes leading to cell death [20]. Moreover, Cell death is induced in a Ca^2+^-dependent or -independent manner, depending on the stimulating agent and cell type [32,33]. Collectively, this study shows that in SW480 cells, [10]-gingerol caused [Ca^2+^]_i_ elevations in a concentration-dependent manner by evoking protein kiase C-independent Ca^2+^ release from the endoplasmic reticulum pathway. These effects may play a crucial role in the physiological action of [10]-gingerol.

## Experimental

### General

Optical rotations were measured with a JASCO DIP-370 digital polarimeter. UV spectra were obtained in MeCN using a JASCO V-530 spectrophotometer. The IR spectra were measured on a Hitachi 260-30 spectrophotometer. ^1^H (400 MHz, using CDCl_3_ as solvent for measurement), ^13^C (100 MHz), DEPT, HETCOR, COSY, NOESY, and HMBC NMR spectra were obtained on a Unity Plus Varian NMR spectrometer. LRFABMS and LREIMS were obtained with a JEOL JMS-SX/SX 102A mass spectrometer or a Quattro GC-MS spectrometer with a direct inlet system. Silica gel 60 (Merck, 230-400 mesh) was used for column chromatography. Precoated silica gel plates (Merck, Kieselgel 60 F-254, 0.20 mm) were used for analytical TLC, and precoated silica gel plates (Merck, Kieselgel 60 F-254, 0.50 mm) were used for preparative TLC. Spots were detected by spraying with 50% H_2_SO_4_ and then heating on a hot plate. 

### Plant Material

The roots of *Zingiber officinale* (ginger) were purchased from a local market of Kaohsiung in Taiwan in July 2006, which was identified by Dr. Yen-Ray Hsui of the Division of Silviculture, Taiwan Forestry Research Institute, Taipei, Taiwan. A voucher specimen (Hsui-Zo-1) was deposited at Fooyin University.

### Extraction and Isolation

The roots (25.6 kg) of *Z. officinale* were chipped and air-dried and extracted repeatedly with CHCl_3_ at room temperature. The combined CHCl_3_ extracts were then evaporated further separated into 20 fractions by column chromatography on silica gel with gradients of *n*-hexane/CHCl_3_. Fr. 9 eluted with CHCl_3_-MeOH (50:1) was next repeatedly subjected to silica gel CC and yielded [10]-gingerol (210 mg) [29].

### Cell Culture and Test Compound Treatment

The SW480 cells were obtained from the American Type Culture Collection. Cells were cultured in Dulbecco's modified Eagle's medium. The media were supplemented with 10% heat-inactivated fetal calf serum, 100 units/mL penicillin and 100 μg/mL streptomycin. Cells were kept at 37 °C in 5% CO_2_-containing humidified air.

### Measurement of [Ca^2+^]_i_

Trypsinized cells (10^6^/mL) were allowed to recover in the culture medium for 1 h before being loaded with 2 μM fura-2/AM for 30 min at 25 °C in the same medium. The cells were washed once with serum-free DMEM medium and resuspended in Ca^2+^-containing medium (pH 7.4) containing (mM): NaCl, 140; KCl, 5; MgCl_2_, 1; CaCl_2_, 2; HEPES, 5; d-glucose, 5. Fura-2 fluorescence measurements were performed in a water-jacketed cuvette (25 °C) with continuous stirring; the cuvette contained 1 mL of medium and 0.5 million cells. Fluorescence was monitored with a Shimadzu RF-5301PC spectrofluorophotometer (Kyoto, Japan) by recording the excitation signals at 340 and 380 nm and the emission signal at 510 nm at 1-sec intervals. Maximum and minimum fluorescence values were obtained by adding 0.1% Triton X-100 and 10 mM EGTA sequentially at the end of each experiment. [Ca^2+^]_i_ was calculated as described previously assuming a *K_d_* of 155 nM [30]. In experiments that were performed in the absence of extracellular Ca^2+^, cells were bathed in Ca^2+^-free medium in which CaCl_2_ (2 mM) was substituted with 0.1 mM EGTA. 

### Cell viability assay

Cell viability was determined by the trypan blue exclusion (TBE) method [31]. Briefly, 10 μL of 4% trypan blue was added to 90 μL of cell suspension, followed by examination with a hemocytometer under an inverted microscope. Cells which excluded the dye were considered viable and the data were expressed as a percentage of that of the corresponding control group. 

### Data analysis

Data is presented as means ± standard deviation (SD) and analyzed using one-way ANOVA with Scheffe′s test. A *p* value of less than 0.05 was considered as statistically significant.
